# Optimization on overall performance of Modified Ultrafine Cementitious Grout Materials (MUCG) and hydration mechanism analysis

**DOI:** 10.1371/journal.pone.0309312

**Published:** 2024-10-03

**Authors:** Junxia Zhou, Lanchang Zha, Shiyu Meng, Yu Zhang

**Affiliations:** 1 Ordos Institute of Liaoning Technical University, Ordos, China; 2 School of Civil Engineering, Liaoning Technical University, Fuxin, Liaoning Province 123000, China; 3 Institute of Engineering Technology, China Construction Sixth Engineering Bureau Corp., Ltd., Tianjin: 300171, China; Shenyang Jianzhu University, CHINA

## Abstract

Given the challenges encountered in injecting grout into micro-cracked rock masses, a modified ultrafine cementitious grout material (MUCG) was developed using ultrafine cement, polyvinyl alcohol (PVA) fibers, and a high-efficiency superplasticizer. To identify the optimal ratio of constituents for grouting these rock masses, extreme difference and multiple linear regression analyses were conducted based on test results of flowability and mechanical properties. A mix comprising 9% silica fume, 0.2% bentonite, 0.3% PVA fibers, 0.15% superplasticizer, and 2% setting accelerator was identified as the optimal mix. The microstructure characteristics of the optimal MUCG (MUCGop) grout cemented body were analyzed using XRD, FTIR, BET, and SEM. XRD and FTIR analyses indicated that a substantial amount of C-(A)S-H gel, CH, and AFt were formed within the first 3 days, highlighting the early strength characteristics of MUCGop. Over time, the content of C-A-H stabilized at 22%, the amount of CH decreased from 19% to 14%, whereas the amount of AFt increased to 15.9% by Day 28. Unexpectedly, CaCO_3_ content increased due to carbonation, reaching 37.3% by Day 28. BET and SEM analyses demonstrated that the specific surface area and porosity (most probable pore size) gradually decreased over time. At various ages, mesopores (cumulative pore diameter, median pore diameter) initially increased and then decreased. Micro-cracks appeared in the cemented body by Day 7, resulting in a slight decrease in strength (3.92%) from Day 3 to Day 7. The formation of well-developed needle-like AFt, C-(A)S-H gel, and small-volume plate-like CH contributed to uniform cementation and a denser structure. From Day 7 to Day 28, there was a slight increase in strength, by an amount of 10.66%. These findings have significant scientific implications for understanding the mechanisms of grouting reinforcement in micro-cracked rock masses.

## 1 Introduction

Underground engineering is characterized by complex hydrogeological conditions, high formation pressures, and susceptibility to disasters such as ground collapse and sudden water inflows during construction disturbances [1]. These issues can be significantly addressed through grouting reinforcement techniques [2]. While grouting technology for large fissured rock masses is relatively mature, addressing micro-cracks presents ongoing challenges [3]. Difficulty in injecting grout can result in insufficient diffusion, reducing the effective bearing area and causing the expansion of micro-cracks [4], thus the structural integrity and mechanical strength of the grout material will be affected [5]. Typically, improving the flowability of the grout can enhance its injectability, but it may lead to poor mechanical properties. The selection of grouting materials and the optimization of comprehensive properties—such as achieving good flowability, low viscosity, and high mechanical strength of the grout [6]—are pivotal for effectively injecting micro-cracks [7] and enhancing mechanical properties and overall structural integrity of materials [5].

In recent years, scholars both domestically and internationally have investigated the incorporation of various additives, including silica fume [8], bentonite [9], and polyvinyl alcohol (PVA) fibers [10], into grout formulations, conducting extensive research on their effects on grout and mechanical properties. Silica fume, with its smaller particles and lighter weight, has been found to effectively fill voids within the cemented body [11]. Researchers like Li et al. [12] have investigated the incorporation of silica fume into solid waste grout materials. Zhang et al. [13] and Hamada et al. [14] incorporated additives, such as silica fume, into cement-based grout materials. Their findings indicate that silica fume reduces porosity and the rate of water bleeding, thereby enhancing the strength and resistance to water dispersion of the grout; however, the incorporation of silica fume increased drying shrinkage. Güllü et al. [15] found that when nano-SiO_2_ was <2.5% and the water/adhesive ratio (w/b) = 1, the fluidity was optimal. Das et al. [16] added silica fume to prepare preplaced aggregate concrete (PAC) and found that the particle size of coarse aggregate did not affect the bond strength (nearly 57%); however, when the compressive strength of conventional concrete and PAC was similar, the bond strength of PAC was significantly lower in comparison to that of conventional concrete.

Bentonite has good lubrication properties, which enhance the flowability of grout; it readily expands upon contact with water, effectively filling the pores in the material [17]. Ma et al. [18] added different concentrations of bentonite to microbial grout. Zheng et al. [19] and Ruan et al. [20] investigated the addition of bentonite to cement-based grout materials. Their studies demonstrated that bentonite enhances fluidity and mechanical strength while reducing the water bleeding rate, shrinkage deformation, and porosity. However, it was noted that high-temperature bentonite may lead to a shorter setting time. Li et al. [21] modified acrylate grout by adding lithium bentonite and found that the lithium bentonite improved the acrylate grout setting time, bleeding, viscosity, slurry retention rate, impermeability, and mechanical strength.

PVA fibers can bridge the cement matrix, form a mesh structure, reduce the formation of micro-cracks, and have high chemical stability, effectively enhancing the mechanical properties and corrosion resistance of materials [22]. Zhang et al. [23] mixed a small amount of PVA fibers into geopolymers; Shen et al. [24] and Wei et al. [25] mixed PVA fibers into cement-based grouting materials. These findings demonstrated that the inclusion of PVA fibers improves compressive, tensile, and flexural properties. Additionally, when arranged in three layers, PVA fibers demonstrated optimal flexural strength. Elmeligy et al. [26] also found that PVA fiber could increase the compressive strength of grouted masonry. Zhu et al. [27] modified cement grout by using carbon fibers, which were found to significantly improve the mechanical properties and crack resistance of specimens. Chen et al. [28] performed erosion and abrasion tests on PVA fiber cement-based composite materials, revealing that PVA fibers offer superior erosion and abrasion resistance. Several other researchers have extensively investigated the performance of fiber-reinforced composite materials in terms of structure, morphology, chemical, and physical properties using various micro-characterization techniques, For instance, Paederia foetida fiber–Al_2_O_3_ powder hybrid-reinforced epoxy composites [29], sugar palm nanofibrillated cellulose (SPNFCs) reinforced sugar palm starch (SPS) composites [30], and PVA agave gigantea cellulose micro fiber (CMF) bio-composite [31] have been extensively investigated. Meanwhile, Ilyas Ra et al. reviewed the development and characterization of natural-fiber-reinforced chitosan, chitosan blends, and their nanocomposites [32]. They examined the distribution within the matrix and interface bonding characteristics using SEM and TEM and optimized the mechanical properties and structural integrity of these fiber composites. XRD patterns demonstrated high crystallinity, which enhances both the mechanical strength and thermal stability of these composites. FTIR analysis demonstrated that the chemical bonds and functional groups at the interfaces, strengthen the interfacial bonding between fibers and matrices, thereby improving the overall mechanical performance of the composites. TGA and DSC analysis indicated good thermal stability and minimal weight loss at high temperatures. Ilyas Ra et al. also studied Eichhornia crassipes (EC) natural fibers, where SEM and FTIR revealed a layered structure on the surface of EC fibers composed of lignin and cellulose. This layered structure imparts flexibility and strength to the fibers. DSC analysis showed that lignin enhances the heat resistance of fibers, suggesting that EC fibers have potential as fillers in composite materials [33]. Hence, these fibers emerge as optimal choices for enhancing the mechanical properties of cementitious grouts. Several researchers have investigated the combined utilization of silica fume, bentonite, and PVA fibers in grout materials. Li et al. [34] enhanced the strength of the cemented body by integrating silica fume and PVA fibers into cement-based grout materials, observing minimal effects on slurry flowability. In their latest study on the mechanical behavior of fiber-reinforced grout (FRG, developed using 15 wt. % silica fume and steel fibers instead of cement), Li et al. found that the minimum volume of fibers capable of enhancing the mechanical properties of the grout is 0.5%. Moreover, when the volume ratio of added fibers reaches 1.5%, the grout achieves its maximum mechanical strength [35], which significantly enhances its ability to prevent crack propagation [36]. Meanwhile, Chen et al. [37] explored the integration of bentonite and fibers into ordinary cement, leading to improved resistance against permeation and cracking. Guo et al. [38] added nano-SiO_2_ and PVA fibers to fly ash-based geopolymers and observed a reduction in setting time, improvement in early compressive strength, and improving the degree of polymerization of amorphous gels. Ashraf et al. [39] enhanced the compressive strength and sulfate resistance of the cemented body while improving the pore structure by substituting a portion of the cement with bentonite and silica fume.

Yu et al. [40] and Kang et al. [41] collectively suggest that ultrafine cement surpasses traditional Portland cement in performance. Its slurry particles are sufficiently small to be effectively injected into microcracks and can substitute toxic and costly chemical slurries. Some scholars have investigated the addition of superplasticizers [42], nano-SiO_2_ [43], and silica fume [44] to ultrafine cement-based grout materials individually. The findings indicate that superplasticizers can enhance fluidity and reduce viscosity but may increase bleeding. Nano-SiO_2_ injection through mesh filters smaller than 30 μm exhibits faster results compared to slot filters for all water/cement ratios. Silica fume enhances compressive and flexural strength but may increase water bleeding. Sun et al. [45] discovered that adding bentonite to the mixture of ordinary cement and ultrafine cement confers anti-leakage properties. Sha F et al. [46] developed a novel type of effective microfine cement-based grout (EMCG) utilizing microfine cement, microfine flue ash (MFA), desulfurization gypsum, and diverse microfine solid wastes. The findings revealed that the inclusion of microfine particles conferred EMCG with a denser pore structure. Moreover, when the naphthalene-based SP (N) content was excessively high, the improvement in fluidity was negligible, thereby increasing the likelihood of a high water bleeding rate. Tian et al. [47] also conducted a similar study, and their results showed that the best fluidity was obtained when the content of ultrafine fly ash (UF) was 40% and that of ultrafine slag (US) was 10%, and that fine particles could effectively fill the pores; in addition, the setting time increased with the increase of the content of the superplasticizer and accelerator.

In summary, the overall performance and high mechanical strength of traditional grouting materials often cannot be balanced simultaneously. Prioritizing multiple slurry properties often leads to diminished mechanical performance. Research on optimizing the overall performance of MUCG and understanding how their hydration mechanisms influence material properties remains limited. This study aims at enhancing strength by partially replacing ultrafine cement with ultrafine silica fume. Additionally, enhanced flowability and stability of the slurry were achieved through the incorporation of bentonite, superplasticizers, and accelerators. The incorporation of ultrafine raw materials and excellent flowability ensures the slurry’s pumpability. Additionally, the addition of PVA fibers and silica fume enhances the overall structural integrity of the grout, improving its compactness and mechanical strength. Based on orthogonal experiments, a formulation of MUCG was developed that simultaneously achieves comprehensive slurry performance (high flowability, low bleeding rate, low viscosity, and setting time) along with high strength. Range analysis, multiple linear regression analysis (MLRA), and optimization objectives were used to determine the optimal mix, designated as MUCGop. Subsequently, XRD was employed to examine changes in the crystalline structure of MUCGop hydration products, and FTIR was employed to detect changes in chemical bonds and functional group absorption peaks. BET was used to measure the material’s pore structure, and SEM analysis was conducted to observe the microstructure and morphology of the material. This comprehensive approach enhanced the understanding of how hydration mechanisms during different curing periods impact the mechanical strength of the material, thereby facilitating further optimization of the material formulation.

## 2 Experimental section

### 2.1 Raw materials

Ultrafine cement (UC) was sourced from Kangcrystal New Materials Technology Co., Ltd. (Shangdong, China); 1000-mesh Lithium-based bentonite (B) was sourced from Shandong Usolf Co., Ltd. (Shangdong, China); 1250-mesh ultrafine silica fume (USF) and PVA fibers were acquired from Shanghai Chenqi Chemical Technology Co., Ltd. (Shanghai, China); Polycarboxylate superplasticizer powder (PSP) was procured from Suzhou FuClear Technology Co., Ltd. (Jiangsu, China); accelerator (Ac) was manufactured by Laiyang Hongxiang Building Additive Factory (Shandong, China). Specific parameters of ultrafine cement and PVA fibers are detailed in Tables 1 and 2, respectively.

**Table 1:** Technical parameters of ultrafine cement.

Cement type	Appearance	Fineness/μm		Flexural strength/MPa		Compressive strength/MPa	
K1340	Grey powder	D50≤5	D90≤10	Day 3≥4	Day 28≥7	Day 3≥23	Day 28n≥52.5

**Table 2:** Technical parameters of PVA fiber.

Length/mm	Density/g/cm3	Tensile strength/MPa	Initial modulus/GPa	Equivalent diameter/μm	Moisture content
3	1.29	1830	40	15.3	<0.1%

**Fig 1:**
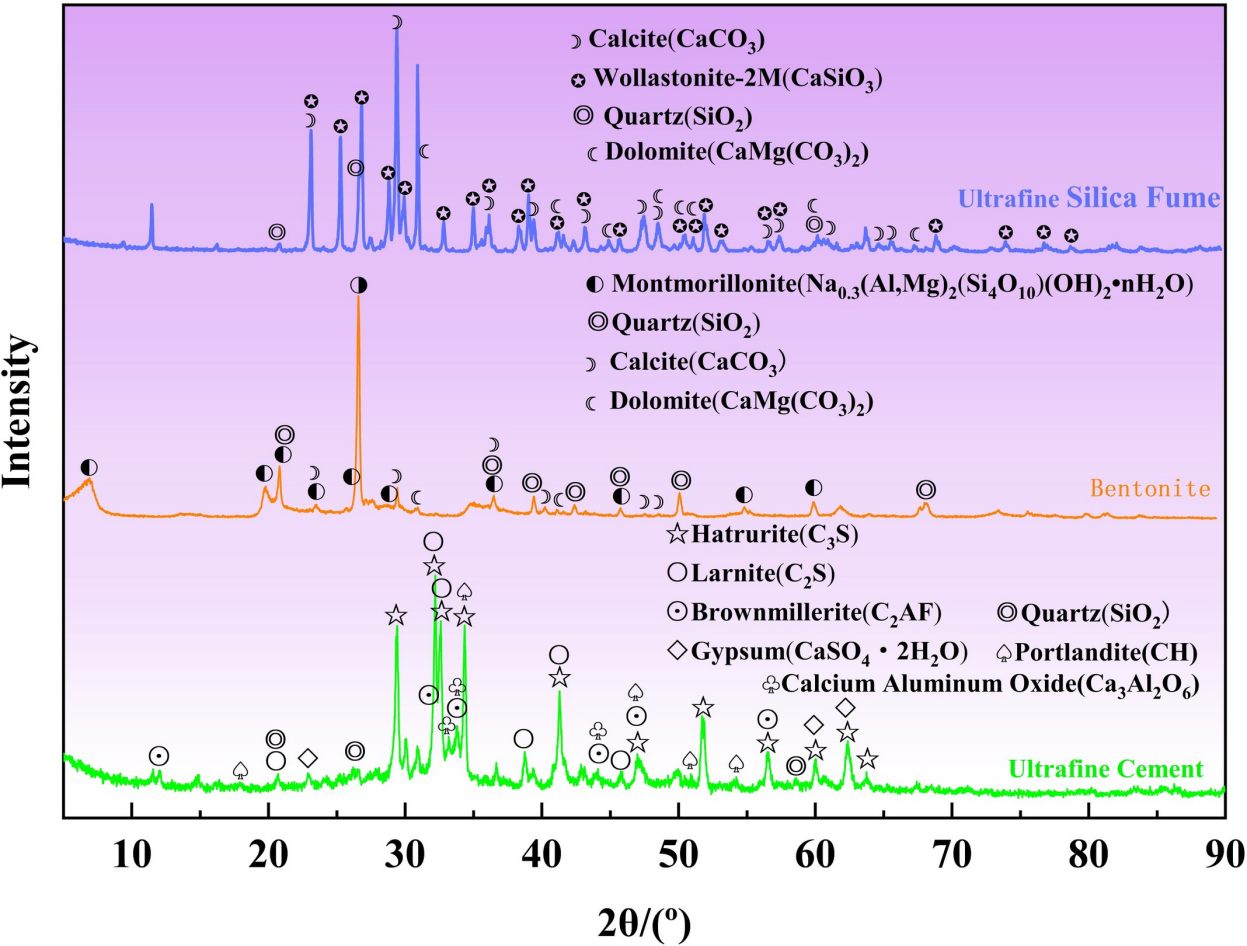
Mineral composition of UFC, B, and SF.

**Fig 2:**
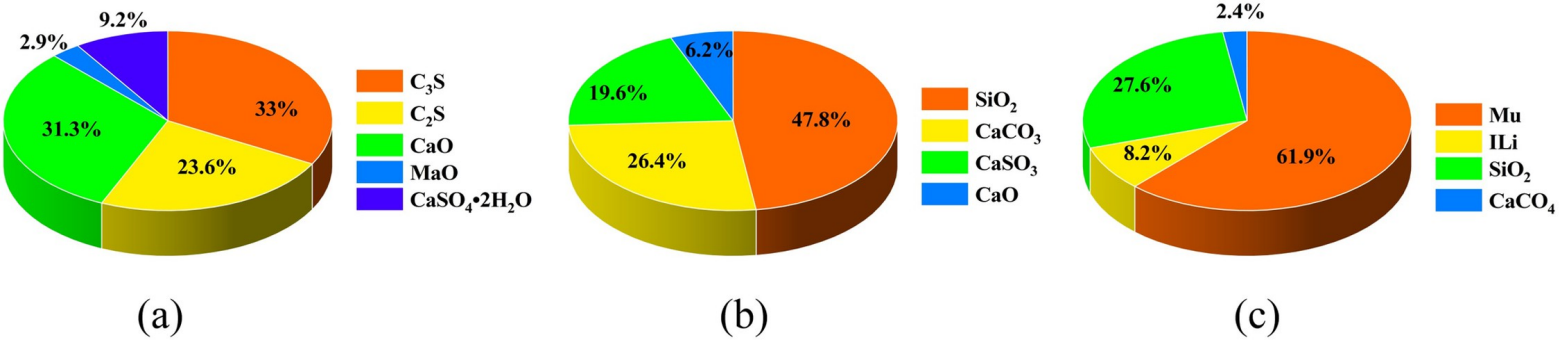
Mineral content of raw materials.

The mineral composition and content of ultrafine cement, lithium-based bentonite, and silica fume were analyzed using XRD, as illustrated in Figs 1 and 2. Superfine cement primarily consisted of C_3_S, C_2_S, C_3_A, C_2_AF, and CaSO_4_•2H_2_O. Lithium-based bentonite was predominantly composed of montmorillonite, SiO_2_, and CaCO_3_. Ultrafine silica powder contains CaCO_3_, CaSO_3_, (quartz) SiO_2_, CaCO_3_, and amorphous SiO_2_ as its main components. It is important to note that the content of amorphous SiO_2_ couldn’t be determined through semi-quantitative analysis.

### 2.2 Specimen preparation

In this experiment, a water-cement ratio of 0.8 was selected [48]. Various mix proportions of MUCG specimens were prepared using the L_16_ (4^5^) Taguchi orthogonal experimental design scheme─This approach minimized the number of experiments required while ensuring the quality and representativeness of the test results [49], as detailed in Table 3, The steps for specimen preparation are as follows:

**Table 3:** Orthogonal design and experimental results for MUCG mix proportions.

Level	Factor					Slurry properties				USB		
	A	B	C	D	E	y1	y2	y3	y4	y5	y6	y7
MUCG_1_	3	0.2	0.1	0.15	0.5	203	54	0	300	7.25	13.94	23.9
MUCG_2_		0.4	0.2	0.2	1	173	33	1.27	269	8.62	12.4	24.86
MUCG_3_		0.6	0.3	0.25	1.5	191	29	2.5	223	9.48	14.16	26.02
MUCG_4_		0.8	0.4	0.3	2	265	24	3.8	186	8.29	15.02	27.43
MUCG_5_	6	0.2	0.2	0.25	2	276	25	4.44	155	9.6	15.17	27.68
MUCG_6_		0.4	0.1	0.3	1.5	265	24	5	235	8.67	14.45	24.89
MUCG_7_		0.6	0.4	0.15	1	163	72	0	254	10.03	16.46	28.52
MUCG_8_		0.8	0.3	0.2	0.5	173	39	0	313	9.63	16.73	28.66
MUCG_9_	9	0.2	0.3	0.3	1	363	23	6.25	262	11.66	18.07	31.78
MUCG_10_		0.4	0.4	0.25	0.5	232	30	2.41	307	12.63	17.94	30.23
MUCG_11_		0.6	0.1	0.2	2	191	35	0	151	9.32	15.76	27.85
MUCG_12_		0.8	0.2	0.15	1.5	181	84	0	206	10.98	17.64	28.27
MUCG_13_	12	0.2	0.4	0.2	1.5	269	36	1.23	199	12.02	16.49	29.21
MUCG_14_		0.4	0.3	0.15	2	207	51	0	136	11.28	18.48	27.97
MUCG_15_		0.6	0.2	0.3	0.5	319	24	3.53	331	10.34	17.16	26.12
MUCG_16_		0.8	0.1	0.25	1	261	33	1.23	271	9.87	14.52	27.74

Note: A∼E represents the content (%) of silica fume, bentonite, PVA fibers, superplasticizer, and accelerator respectively; y1∼y7 represent the results of flowability (mm), viscosity (s), water bleeding rate (%), setting time (min), and compressive strength (MPa, Day 3, Day 7, Day 28).

**Fig 3:**
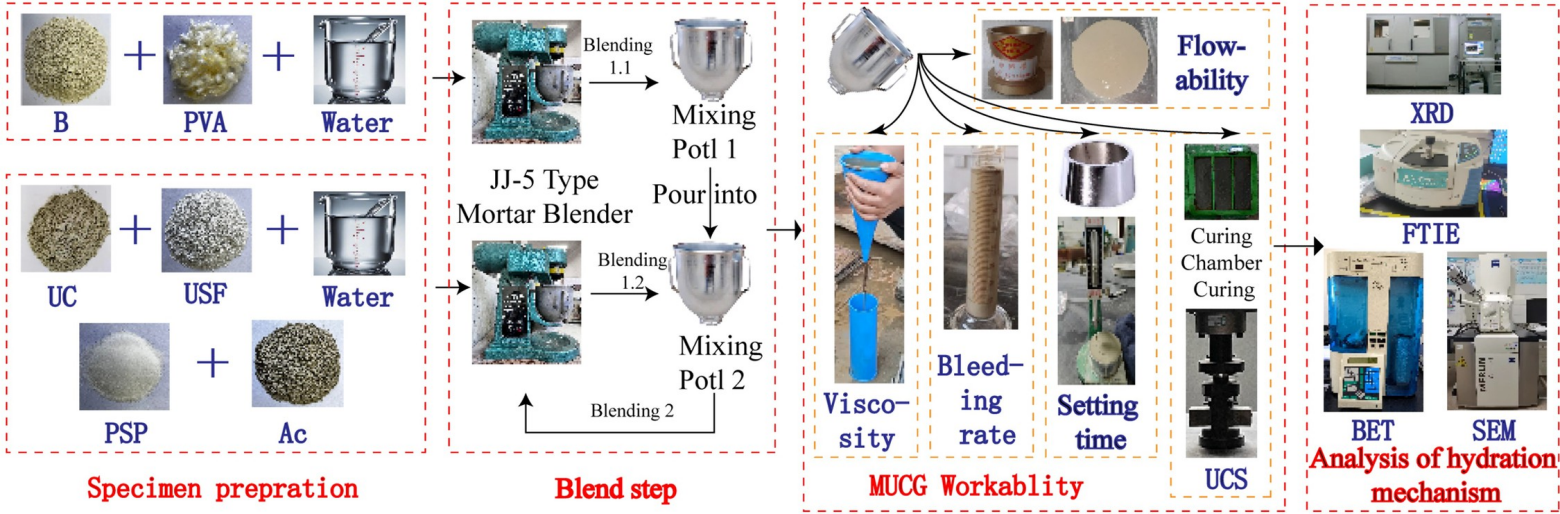
Sample preparation and testing flow chart.

Fist, due to the hydrophilicity of PVA fibers and the water-expanding property of lithium-based bentonite, these two components were first added to mixing pot 1 and thoroughly mixed. Subsequently, measured amounts of ultrafine cement, silica fume, superplasticizer, and accelerator were added to mixing pot 2 and mixed. Both mixing pots were then placed in the cement mortar mixer sequentially and stirred at medium and low speeds for 120 s. The contents of mixing pot 1 were then poured into mixing pot 2, followed by high-speed stirring for an additional 60 s. Second, the flowability, viscosity, water bleeding rate, and setting time were tested successively according to the experimental specification in section 1.2. The resulting slurry was then poured into tri-molds with dimensions of 40 mm × 40 mm × 160 mm and cured under standard conditions (the curing box was equipped with sensors to monitor and adjust the temperature and humidity in the box in real-time and ensured that the indicators remained within the required standard range: temperature 20 ± 1°C, humidity > 95%) for 24 h. After standard curing periods of 3, 7, and 28 days, unconfined compression strength tests were conducted. Subsequently, MUCGop was analyzed using XRD, FTIR, BET, and SEM in sequence. The sample preparation and related testing process are illustrated in Fig 3.

### 2.3 Experimental methods and instruments

Flowability testing was conducted following the specifications outlined in " GB/T 8077–2012 Methods for Testing Uniformity of Concrete Admixtures." The test utilized a truncated cone mold (upper diameter: 36 mm, lower diameter: 60 mm, height: 60 mm, made of seamless metal with smooth inner walls) for measurements Before the initiation of the experiment, the instrument was wiped following the specification. The truncated cone mold was placed in the center of the 400 mm × 400 mm × 5 mm glass plate. The slurry was then quickly injected into the truncated cone mold, and the surface was smoothed with a scraper. Subsequently, the truncated cone mold was lifted vertically upward, and after the passage of 30 s, a 300 mm steel ruler was used to measure the maximum diameters of the flowing parts in both directions perpendicular to each other. The average of both measurements was taken as the fluidity.

A type 1006 Marsh funnel viscosimeter was employed to determine the apparent viscosity by measuring the time it took for 700 mL of slurry to flow out 500 mL from the funnel.

Additionally, 100 mL of the slurry was poured into a 100 mL graduated cylinder with a stopper (graduation value 1 mL), and the water bleeding rate was determined by the ratio of the height of the water layer H_2h_ observed on the surface of the slurry after 2 h of settling to the initial height of the slurry H_0_. The static environment was a stable curing room at a temperature of 20±1°C and a simulated indoor relative humidity of 35±5%. The temperature during the sedimentation was controlled to reduce the influence of external vibration on the slurry sedimentation.

The setting time test was conducted following the specifications outlined in "GBT 1346–2011 Test Methods for Water Requirement of Normal Consistency, Setting Time, and Soundness of the Portland Cement" using a Vicat apparatus. The curing and testing interval of specimens were adjusted following the specification. The test mold was removed and placed under the ring test needle. The screw was tightened, and after 2 s, the needle was released suddenly. The test needle sank vertically and freely into the slurry. When the needle left no mark on the specimen, the pointer reading at that moment represented the final setting time of the slurry. This final setting time served as the benchmark for setting time in this study.

Unconfined compressive strength testing adhered to the "GB/T 17671–2021 Test Method of Cement Mortar Strength (ISO Method)" and was performed with a WDW-100E microcomputer-controlled electronic universal testing machine from Jinan Times Testing Instrument Co., Ltd. The load was uniformly applied at a force control rate of 0.05N/s and a displacement rate of 0.5 mm/min. The compressive strength (Rc) in megapascals (MPa) was calculated as the ratio of the maximum load (Fc) in newtons (N) to the compressive area (A) of 1600 mm² at the point of fracture. Unconfined uniaxial compressive strength tests were conducted on the Day 3, 7, and 28, and the average of three specimens per group was taken (subscripts were taken if the error exceeded 15%, specifically, if one of the three strength values exceeds the average value by 10%, it should be excluded, and the remaining two values are averaged to determine the bending strength test result; if two of the three strength values exceed the average by 10%, the remaining value is considered the measured compressive strength).

The samples were dried, ground, sieved through a 200-mesh sieve, and then subjected to XRD and FTIR analyses. BET and SEM samples were prepared as thin slices. The mineral composition and content of raw materials and optimal specimens were determined using an X-ray diffractometer (Rigaku D/MAX-2600, Japan), with a scanning angle range of 5∼90° and a scanning speed of 2°/min. The chemical bond characteristics of the hydration products were analyzed using a Thermo Scientific Nicolet iS20 Fourier Transform Infrared (FTIR) spectrometer, over a wave velocity range of 400–4000 cm^-1^. The Quantachrome Nova 4000e Autosorb IQ Brunauer–Emmett–Teller (BET) from the United States was employed for nitrogen degassing and specific surface area and pore volume analysis (including specific surface area and pore size distribution, including mesopores and micropores), with a degassing temperature of 200°C, and a synthesis temperature of 500°C. The microstructure of the optimal specimens was observed using a scanning electron microscope (SEM) (Zeiss Merlin CoMPact, Germany), utilizing backscatter electron (BSE) imaging mode.

## 3 Results and analysis

### 3.1 Significance analysis of influencing factors

#### 3.1.1 Range analysis

**Fig 4:**
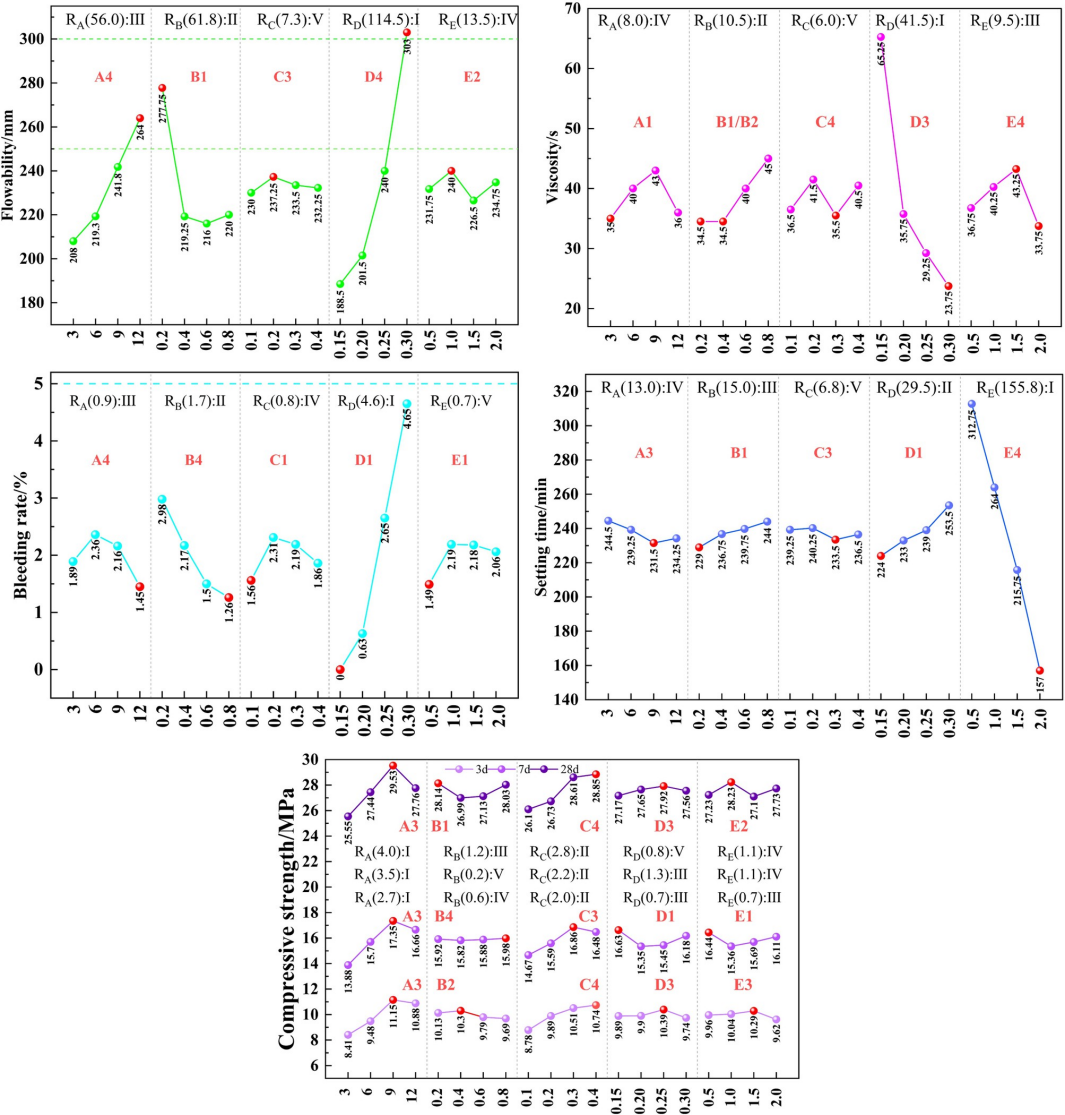
Normalization of MUCG properties of slurry and UCS values.

To elucidate the correlation between different levels of various factors and the properties as well as the compressive strength of the slurry, averaging and range ranking of the MUCG slurry properties and UCS test results in Table 3 were performed. These findings are depicted in Fig 4 (take the fluidity in Fig 4 for example, "RA(56.0) III" means that the range of factor A is 56.0, which ranks third among the five factors).

According to Fig 4, the order of the factors influencing flowability, viscosity, water bleeding rate, setting time, and compressive strength (Day 3, Day 7, and Day 28) in the range analysis is as follows:

(i) Flowability: R_D_(114.5) >R_B_(61.8)> R_A_(56.0)> R_E_(13.5) >R_C_(7.3). The superplasticizer had the most significant influence on the slurry’s flowability, followed by silica fume and bentonite. The addition of superplasticizer and silica fume significantly increased the flowability by 114.5 mm and 56.0 mm respectively. With the increase in bentonite content from 0.2% to 0.4%, the flowability decreased significantly by 58.5 mm and stabilized when bentonite content exceeded 0.4%. (ii) Viscosity: R_D_(41.5) >R_B_(10.5)> R_E_(9.5)> R_A_(8.0) >R_C_(6.0). For viscosity, the superplasticizer also had the most substantial impact, with bentonite exerting a slightly greater influence than the other factors. As the superplasticizer content increased, the viscosity significantly decreased by 41.5 s. Conversely, an increase in bentonite content resulted in a viscosity increase of 10.5 s. (iii) Water bleeding rate: R_D_(4.6) >R_B_(1.7)> R_A_(0.9)> R_C_(0.8) >R_E_(0.7). The superplasticizer had the most significant effect on the water bleeding rate, followed by bentonite, while the other three factors had a less noticeable impact.

The reasons for phenomena (i), (ii) and (iii) are as follows: The increase in superplasticizer content resulted in a significant 4.6% increase in water bleeding rate. Conversely, increasing the bentonite content decreased the water bleeding rate by 1.7%. This phenomenon occurs because both polycarboxylate ether (PCE) superplasticizer molecules and bentonite particles carry negative charges, which interact with the positively charged cement particles; this interaction helps to disperse cement particles, reducing their aggregation [50]. The long side chains of PCE form physical barriers between cement particles and increase the interlayer spacing when bentonite absorbs water, thus reducing friction between cement particles [51]. Moreover, the synergistic action of PCE molecules and bentonite significantly enhances the flowability and reduces the viscosity of the slurry. Additionally, PCE effectively disperses cement particles, allowing the tiny particles of silica fume to better fill the spaces between the cement particles. This reduces internal friction and further promotes the dispersion of cement particles by PCE. The mutual enhancement between PCE and silica fume thus significantly improves flowability [52]. Meanwhile, the combined effect of bentonite’s water absorption and PCE’s dispersibility stabilizes the slurry structure, reducing the bleeding of free water and lowering the water bleeding rate [53].

(iv) Setting time: R_E_(115.8) >R_D_(29.5)> R_B_(15.0)> R_A_(13.0) >R_C_(6.8). The accelerator had the greatest impact on setting time, with the superplasticizer being the second most significant factor; the remaining factors had minimal effects. Increasing the content of the accelerator significantly shortened the setting time by 155.8 min, while increasing the superplasticizer content extended the setting time by an additional 29.5 min. This is because the accelerator accelerates the initial hydration reaction of cement by providing additional reaction sites and promoting the formation of ettringite, thereby significantly reducing the initial setting time [54]. The electrostatic repulsion of the PCE superplasticizer disperses the cement particles, while the long side chains of the PCE create steric hindrance between the particles. This combination slows down the hydration reaction and prolongs the setting time [55].

(v) Compressive strength (at 3, 7, and 28 d): R_A_(4.0) >R_C_(2.8)> R_B_(1.2)> R_E_(1.1) >R_D_(0.8) at 3 d, R_A_(3.5) >R_C_(2.2)> R_C_(2.2)> R_D_(1.3) >R_E_(1.1) at 7 d, R_A_(2.7) >R_C_(2.0)> R_D_(0.7) = R_E_(0.7) >R_B_(0.6) at 28 d. In terms of compressive strength on Days 3, 7, and 28, silica fume had the most significant impact, followed by PVA fibers, with the other factors having minimal effects. The compressive strengths at 3d, 7d, and 28d increased by 2.7 MPa, 3.5 MPa, and 4.0 MPa, respectively, as the silica fume content increased from 3% to 9%. However, the compressive strengths decreased when the silica fume content exceeded 9%. With increased PVA fiber content, the compressive strengths at 3d, 7d, and 28d rose by 2.0 MPa, 2.2 MPa, and 2.8 MPa respectively. This phenomenon occurs because silica fume reacts with Ca(OH)_2_ produced by hydration of cement to form C-S-H gel, and together with the unreacted ultrafine silica fume powder, fills the pores of the cement body, thus improving the compressive strength [56]. PVA fibers mechanically link the hydration products among the raw materials, creating a cohesive structure that enhances the mechanical strength of the cemented grout body [57].

#### 3.1.2 Multiple Linear Regression Analysis (MLRA)

The results of the range analysis provide an intuitive understanding of the significance of each factor on the material properties, but they cannot accurately quantify this significance. To address this limitation, MLRA was conducted on the experimental results in Tables 4 and 5 to establish regression models, as shown in Eqs (1) to (7).


y1=134.625+19.05A−17.65B+0.3C+38.20D−0.45E (1.)

y2=60.5+0.6A+3.7B+0.6C−13.1D−0.6E (2.)

y3=−0.834−0.138A−0.583B+0.079C+1.596D+0.172E (3.)

y4=344−3.85A+4.8B−1.5C+9.45D−51.55E (4.)

y5=6.718−0.907A−0.183B+0.652C+0.006D−0.078E (5.)

y6=12.15+1.0A+0.025B+0.670C−0.126D−0.068E (6.)

y7=22.454+0.872A−0.021B+1.013C+0.144D+0.039E (7.)

**Table 4:** Description of fitting models.

						F		
Model	R	R^2^	Sum of	Degrees of	Mean	F-0.05	Sig.	
			squares	freedom	square			Note
y1	0.922	0.851	42679.15	5	8535.83	11.378	<0.001	***
y2	0.874	0.763	3727.6	5	745.52	6.447	0.006	**
y3	0.953	0.909	58.806	5	11.761	19.865	<0.001	***
y4	0.997	0.994	55736.35	5	11147.27	306.749	<0.001	***
y5	0.902	0.813	25.742	5	5.148	8.688	0.002	**
y6	0.8	0.64	29.385	5	5.877	3.549	0.042	*
y7	0.759	0.576	36.188	5	7.238	2.713	0.084	/

**Table 5:** Results of significance for regression factors.

		A	B	C	D	E
y1	P	0.011	0.016	0.962	<0.001	0.943
	Sig.	*	*	/	***	/
y2	P	0.808	0.155	0.808	<0.001	0.808
	Sig.	/	/	/	***	/
y3	P	0.441	0.007	0.658	<0.001	0.341
	Sig.	/	**	/	***	/
y4	P	0.017	0.005	0.092	<0.001	<0.001
	Sig.	/	/	/	***	***
y5	P	<0.001	0.313	0.004	0.972	0.661
	Sig.	***	/	**	/	/
y6	P	0.006	0.933	0.042	0.67	0.817
	Sig.	**	/	*	/	/
y7	P	0.038	0.955	0.02	0.701	0.918
	Sig.	*	/	*	/	/

Table 4 presents the description of fitting models y1 to y7. The closer the R-value of the Correlation Coefficient is to 1, the more accurate the factor is in explaining the variation of the response variable; if the R^2^-values of the Coefficient of Determination are all close to 1, it points to the ability of the model to account for all variations of the response variable the accurately [58]. A larger Regression Sum of Squares (SSR) and Mean Square Regression (MSR) indicate that the model explains more variability, that each independent variable has stronger explanatory power for the dependent variable, and that the model has better predictive ability. The degrees of freedom for regression (df_Regression or df_R) refer to the number of independent variables. A larger F value corresponds to a smaller p-value, indicating that the multiple linear regression model is more significant [58].

From Table 4, the correlation coefficients between the dependent variables y1 to y7 and the independent variables A to E are 0.922, 0.874, 0.953, 0.997, 0.902, 0.800, and 0.759, respectively, with R-value close to 1 indicating that this factor can well explain the change of the response variable. The fitted linear Coefficient of Determination is 0.851, 0.763, 0.909, 0.994, 0.813, 0.604, and 0.576, respectively; the R^2^-values of y1∼ y5 are all close to 1, which implies that the model can well explain all the variation of response variables. The SSR and MSR-values of y1 to y7 both are larger, indicating that the model fits well. Models y1 to y6 are all significant, while y7 is very close to being significant, indicating a high level of statistical significance for this model. The R^2^-value for y6 and y7 is low, and the P-value for y7 is 0.084, which exceeds 0.05. This suggests that the actual relationship may be nonlinear and that multiple linear regression analysis (MLRA) does not effectively predict y6 and y7. Li et al. confirmed this observation [59]. This is attributed to the decreasing consumption of silica fume in the raw materials as the curing time lengthens, resulting in a diminishing impact on compressive strength [56].

Table 5 shows the significance of factors in the multiple linear regression analysis (MLRA). A larger absolute value of the t statistic (or one closer to zero) indicates a more significant (or insignificant) impact of the independent variable on the dependent variable. A smaller p-value associated with the t statistic signifies a greater significance of the factor [60]. The relative influence of the parameters can be assessed through the weighting of p-values [49]. *: Significant at P < 0.05; **: Highly significant at P < 0.01; ***: Extremely significant at P < 0.001; /: Not significant at P > 0.05.

According to Table 5, the significance of factors identified in MLRA for flowability, viscosity, water bleeding rate, setting time, and compressive strength (Day 3, Day 7, and Day 28) are as follows:

(i) Flowability: It demonstrates that the superplasticizer had an extremely significant impact on flowability, while silica fume and bentonite had a significant effect on flowability as well. (ii) Viscosity: D(***)>B(/) = E(/) = A(/) = C(/). Moreover, the superplasticizer demonstrated an extremely significant influence on viscosity. (iii) Water bleeding rate: D(***) >B(**)> A(/) = C(/) = E(/). Similarly, the superplasticizer significantly affected the water bleeding rate, while bentonite also had a highly significant impact on this parameter. (iv) Setting time: E(***) = D(***)>B(/) = A(/) = C(/). Furthermore, both the accelerator and superplasticizer exhibited an extremely significant effect on setting time. (v) Compressive strength (at 3, 7, and 28 d): A(***) >C(**) = B(/) = E(/) = D(/) at 3 d, A(**) >C(*)> C(/) = D(/) = E(/) at 7 d, (*) >C(*)> D(/) = E(/) = B(/) at 28 d. In terms of compressive strength on Day 3, silica fume had an extremely significant influence, whereas PVA fibers showed a highly significant impact on this parameter as well. On Day 7, silica fume also had a highly significant impact on the compressive strength, while PVA fibers had a significant effect. Both silica fume and PVA fibers were found to have a significant influence on the compressive strength on Day 28.

**Fig 5:**
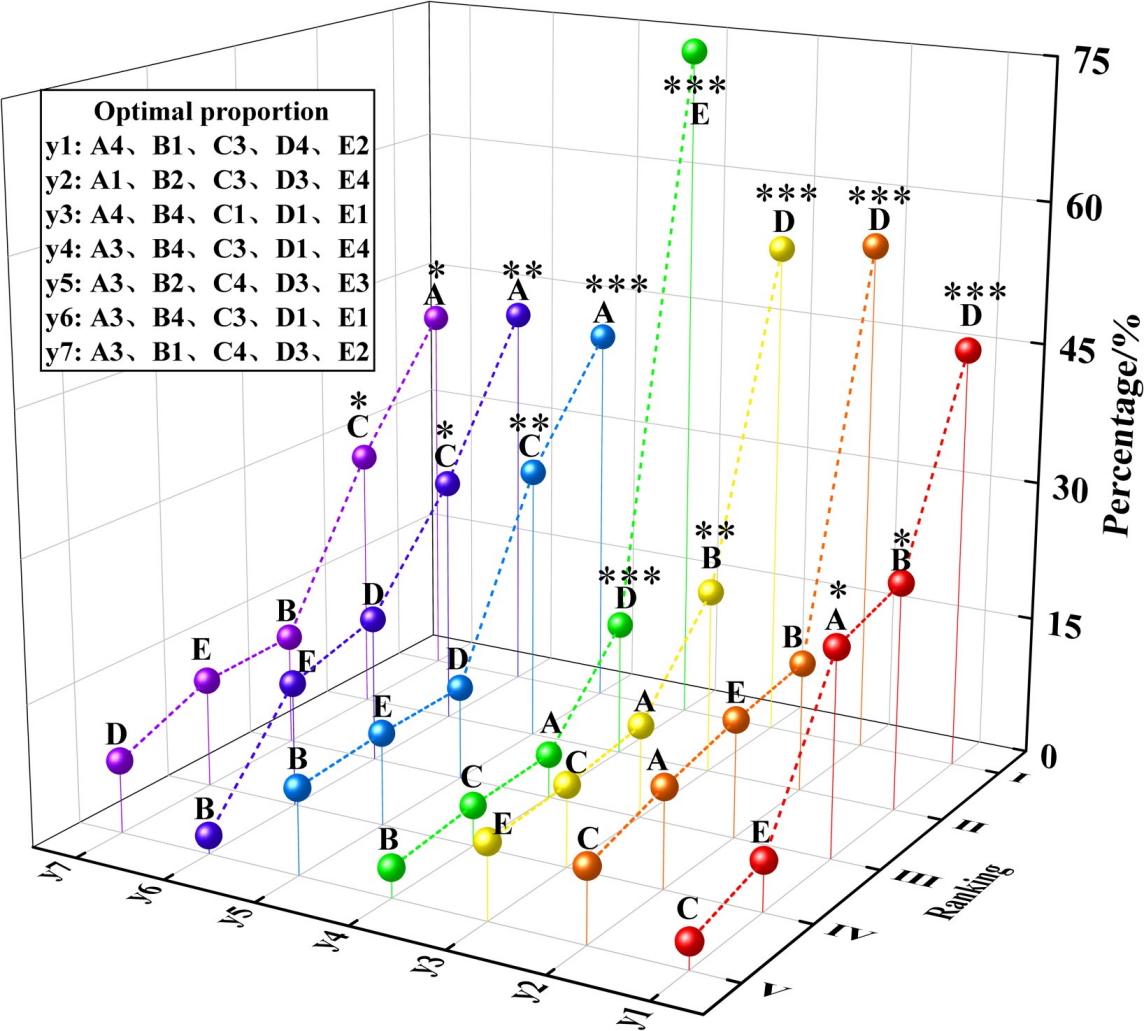
Significant condition.

In Fig 4, the range of differences was standardized in descending order (standardization: the percentage of the range value of a certain factor to the total range of the five factors), and the optimal proportions (detailed in Section 2.3) were summarized in a table. According to Table 5, the significance of regression factors was annotated, as illustrated in Fig 5 The results demonstrate a high consistency between the significance of factors identified in MLRA and those identified in the range analysis, as follows:

(i) Flowability: R_D_(114.5&***)>R_B_(61.8&*)>R_A_(56.0&*). (ii) Viscosity: R_D_(41.5&***). (iii) Water bleeding rate: R_D_(4.6&***) >R_B_(1.7&**). (iv) Setting time: R_E_(115.8&***) >R_D_(29.5&***). (v) Compressive strength (Day 3, Day 7 and Day 28): R_A_(4.0&***) >R_C_(2.8&**), R_A_(3.5&**) >R_C_(2.2&*) and R_A_(2.7&*) >R_C_(2.0&*).

### 3.2 Evaluation criteria

The slurry for grouting in micro-cracked rock masses must meet the following requirements:

Within 30 min, the flowability should range between 250 to 300 mm, and the water bleeding rate should not exceed 5%. This ensures the slurry can effectively diffuse into the micro-cracks of the rock mass and meets the requirements for slurry pumping during construction [61]. To ensure better penetration into micro-cracks, lower viscosity is preferred, but it should not fall below the minimum limit of 18.67 s for funnel viscosity [48]. The lowest value within the viscosity range of 23 to 65 s should be used as the evaluation standard to satisfy both penetration and handling requirements. The setting time should allow sufficient penetration of the slurry into micro-cracks without being excessively long, ensuring that the strength characteristics are quickly developed to enhance the reinforcement effect on the rock mass [62]. The lowest value within the setting time range of 160 to 310 min should be used as the evaluation standard to meet both requirements. The Day 28 compressive strength of the cemented body should exceed 17 MPa [63]. Using the highest value within the compressive strength range as the evaluation standard is anticipated to enhance the bearing capacity of the rock mass.

### 3.3 Optimal proportions of individual materials

Based on section 2.2, the optimal levels of certain factors in Fig 4 were analyzed, with the red nodes representing the optimal levels. This analysis allowed for the determination of the optimal proportions of individual materials: A1 and B1 fell within 250 to 300 mm, and C2, D4, and E2 were closest to 250 to 300 mm, thus constituting the optimal flowability ratio of A1, B1, C2, D4, E2. The lowest viscosity was associated with the levels A1, B2, C3, D4, and E4. The water bleeding rate consistently stayed below 5%, with the optimal levels being A4, B4, C1, D1, and E1. The shortest setting time corresponded to the levels A3, B4, C3, D1, and E4. The highest compressive strength on Days 3, 7, and 28 was achieved with the combinations A3, B2, C4, D3, E3; A3, B4, C3, D1, E1; and A3, B1, C4, D3, E2, respectively.

### 3.4 Optimal proportion analysis

#### 3.4.1 Comprehensive balance analysis of optimal proportions

**Fig 6:**
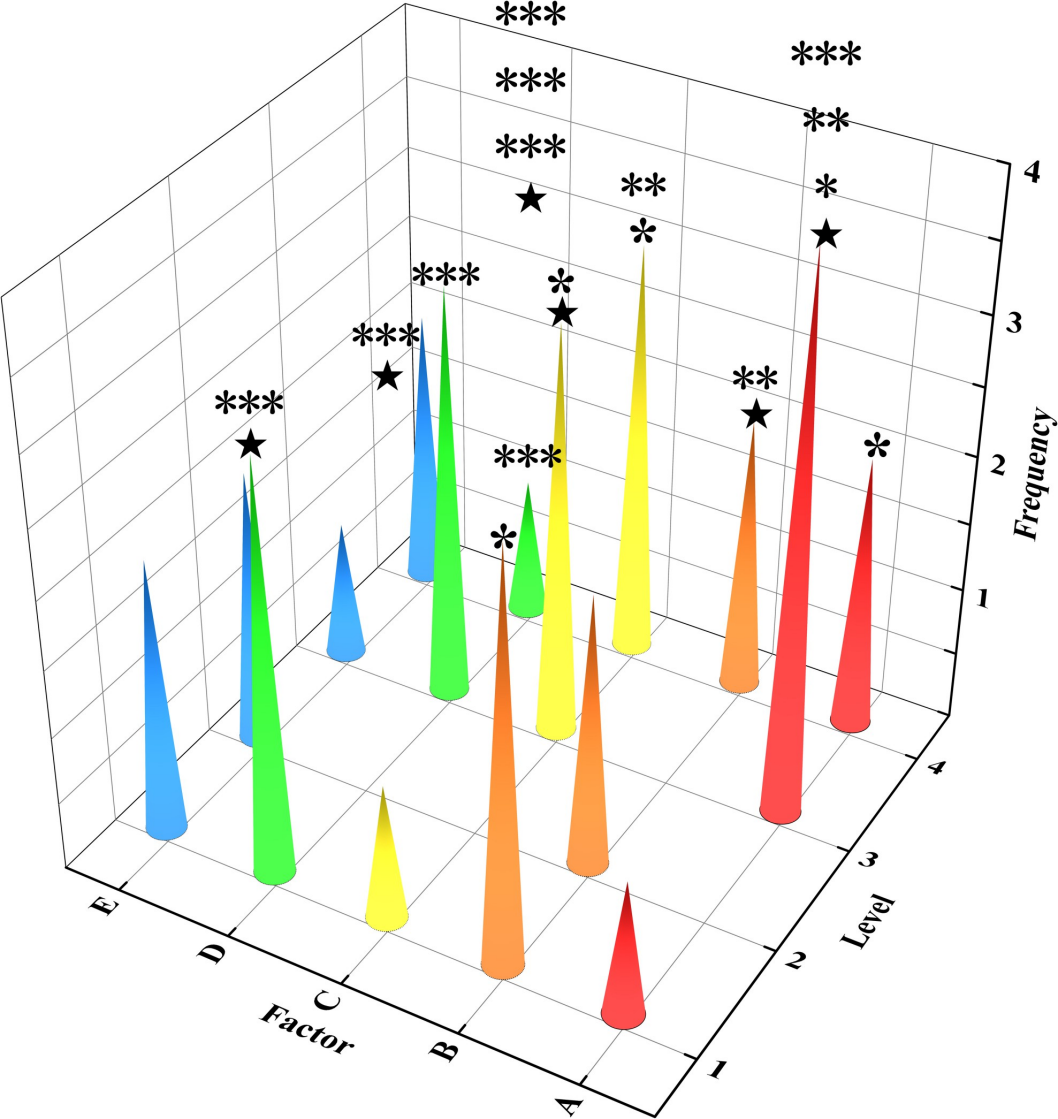
Optimal proportion under comprehensive balance analysis (vertical "*" indicates the number of occurrences for a certain factor at a certain level, horizontal "*" denotes the degree of significance, "★" signifies the optimal proportion).

Fig 6, the z-axis represents the frequency of a certain factor at a specific level, obtained through statistical analysis of the optimal proportion data in Fig 5, with significance annotations also derived from Fig 6. Based on the “2.2 Evaluation Criteria”, which emphasize the comprehensive performance of slurry, the significance annotations in as primary criteria, and the frequency of a certain level under a particular factor as secondary criteria, the final optimal ratios were determined as follows:

A3 appears four times, with three of these instances being significantly above, while A4 appears twice, with only one of these being significant. This indicates that A3 is the optimal level;

B1 appears three times, with one occurrence being significant. B2 and B4 each appear twice, with B4 having one extremely significant occurrence. However, B1’s balanced occurrences in flowability, setting time, and compressive strength make it the optimal level;

Both C3 and C4 appear twice, with C3 having one significant occurrence and C4 having one highly significant and one significant occurrence. C3’s occurrences are evenly distributed across viscosity, setting time, and compressive strength, whereas C4’s occurrences are limited to viscosity and compressive strength. Therefore, C3 is considered the optimal level;

D1 and D3 both appear thrice, with 1 occurrence being extremely significant for both; D1’s occurrences are evenly distributed among water bleeding rate, setting time, and compressive strength, while D3 is observed only in viscosity and compressive strength, leading to D1 being the optimal proportion;

E1, E2, and E4 each appear twice, but only E4 has an extremely significant occurrence, while the occurrences for E1 and E2 are not significant. Therefore, E4 is chosen as the optimal level. The final optimal proportion is a combination of A3, B1, C3, D1, and E4.

#### 3.4.2 Performance comparison between MUCG_OP_ and other grouting materials

The best MUCG performance was tested according to the optimal proportion of A3 (9%), B1 (0.2%), C3 (0.3%), D1 (0.15%) and E4 (2%). The results are presented in Table 6.

**Table 6:** Slurry performance and compressive strength of MUCG and other grouting materials.

Grouting materials	Mix proportion	W/C	Flowability /mm	Viscosity/s	Bleeding rate/%	Seting time	Compressive strength/MPa		
							3d	7d	28d
MUCG		0.8	262	24.53	1	168 min	17.58	16.89	18.69
C-CG [64]	P.O 42.5:B = 80:20	0.45	/	6	/	375 min	5∼7.5	5∼7.5	10
GGM [64]	{(FA:S = 2:3)+[NaOH+SiO_2_ (29.7): Na_2_O (9.8)]}:B:LP = {75}:20:5	0.7	/	9	/	325 min	1.4	1.5∼2.5	1.5∼2.5
SCGPop [19]	P.O 42.5:B:FA:Ret = 60:30:30:0.45	1:1	148	/	2.41	60.1 h	/	1.2	9.8
C-S [65]	[P.O 42.5(0∼100,intervals of 10)+SF(0∼40,intervals of 10)+HEMC(0∼1,intervals of 0.5) ]:Sodium Silicate Solution = [50:50]	1:1	170, 10%SF; 250, 0.5HEMC.	/	/	40 s, 10%SF; 30 s, 0.5HEMC.	/	/	2, 10%SF; 4.5, 0.5HEMC.

Notes:① C-CG: clay-cement composite grouting material; GGM: geopolymer grouting material; SCGPop: slow-setting cement-based grouting paste optimal ratio; C-S: cement-sodium silicate grout. ② P.O 42.5: ordinary portland cement 42.5; B: bentonite; FA: Fly ash; S: Slag; LP: limestone powder; Ret: retarder; HEMC: hydroxyethyl methyl cellulose. ③ The “viscosity” data for C-CG and GGM are referred to as “flowability” in the original paper. However, since the experimental procedures for obtaining these data are identical to those used in this study, they are also considered viscosity.

Based on Table 6, the following conclusions can be drawn: (i) MUCGop demonstrates superior flowability compared to SCGP, C-S (10% SF), and C-S (0.5% HEMC), with improvements of 114 mm, 92 mm, and 12 mm, respectively. MUCGop’s enhanced flowability makes it more suitable for injecting into micro-cracks. In contrast, SCGP and C-S exhibit lower flowability, resulting in incomplete grouting and backflow issues [66]. (ii) MUCGop’s viscosity is 18.53 s and 15.53 s higher than C-CG and GGM, respectively. MUCGop’s viscosity is adequately high, indicating good slurry cohesion. In contrast, C-CG and GGM exhibit excessively low viscosity, resembling water-like states with poor cohesion, which is detrimental to effective crack filling [67]. (iii) MUCGop’s water bleeding rate is 1.41% lower than that of SCGPop, indicating a more stable slurry that is less likely to segregate during pumping [68]. (iv) Setting time varies significantly depending on specific research objectives. (v) MUCGop’s compressive strengths at 3d, 7d, and 28d significantly surpass those of C-CG (10.08 MPa, 9.49 MPa, and 8.69 MPa) and GGM (16.18 MPa, 14.39 MPa, and 16.19 MPa). Additionally, MUCGop’s compressive strengths at 7d and 28d exceed those of SCGPop (15.69 MPa and 8.89 MPa), and its 28-day strength is notably higher than that of C-S (10% SF, 0.5% HEMC) (16.69 MPa and 14.19 MPa). Overall, MUCGop demonstrates superior compressive strength. (3.8)

A comparative evaluation of MUCGop, C-CG, and other advanced grouting materials (GGM, SCGPop, and C-S) in terms of cost-effectiveness, usability, and long-term performance led to the following conclusions:

- MUCGop, which utilizes ultrafine cement and additives, results in higher material costs and requires specialized preparation. Despite these factors, it excels in overall performance and strength, offering excellent injectability and durability, making it ideal for grouting projects that demand high injectability and strength.
- C-CG substitutes ordinary cement with bentonite, leading to lower costs. However, its slurry performance and strength are inferior and sensitive to environmental conditions. Despite being easy to use, it is more suitable for large-scale grouting projects [69].
- GGM further replaces ordinary cement with solid waste slag based on C-CG. While it offers lower costs and environmental benefits, its slurry performance under a water-cement ratio of 0.7 is not significantly improved, and its strength is lower than that of C-CG. Adjusting the water-cement ratio can enhance its strength for specific grouting projects, although it is slightly more complex to use compared to C-CG. It holds potential for broader application [70].
- SCGPop substitutes ordinary cement with bentonite and fly ash, providing lower costs and good slurry performance. However, its strength is average, and it requires a more complex operation. The addition of retarders is beneficial for projects requiring repeated grouting, but it may be less suitable for other types of grouting applications [71].
- C-S substitutes ordinary cement with SF, added with HEMC and mixed with sodium silicate solution (1:1). This results in higher costs but provides rapid setting and initial strength. However, C-S shows significantly lower compressive strength underwater compared to its strength in air, indicating poor adaptability to water environments. The extensive use of sodium silicate solution poses environmental contamination risks. Moreover, preparing C-S requires standardized operations by professionals, limiting its use [72]. Thus, aside from C-CG, which has broad applicability, other grouting materials are suited for specific environmental engineering applications.

In summary, the excellent fluidity (262 mm) and low viscosity (24.53 s) of MUCGop confer good injectivity to the slurry within micro-cracks. A moderate setting time (168 min) allows ample time for the slurry to diffuse within micro-cracks. A 1% water-bleeding rate facilitates minimal segregation during slurry pumping. Moreover, high compressive strength ensures effective grouting reinforcement. Thus, while MUCGop incurs unavoidably higher costs and operational complexity during production and use, its excellent overall performance and high mechanical strength are invaluable for underground engineering grouting projects requiring good injectability and high load-bearing capacity.

With MUCGop’s optimized properties established, the next crucial step involves analyzing the temporal evolution of its compressive strength to inform future improvements. The Day 3 compressive strength of MUCGop exceeded the Day 28 compressive strength requirement, indicating that MUCGop has early strength characteristics. The compressive strength showed a slight decline on Day 7 and a minor increase on Day 28. Specifically, there was a 3.92% decrease from Day 3 to Day 7, a 6.31% increase from Day 3 to Day 28, and a 10.66% increase from Day 7 to Day 28. To further understand the early strength and macroscopic compressive strength changes of MUCGop, it was essential to investigate the micro-hydration mechanism of MUCGop at different curing ages.

### 3.5 Micro-hydration mechanism analysis of cemented body

#### 3.5.1 Hydration product analysis by XRD

The hydration products and contents of MUCGop at different curing ages were analyzed by XRD to investigate the variation law of compressive strength.

**Fig 7:**
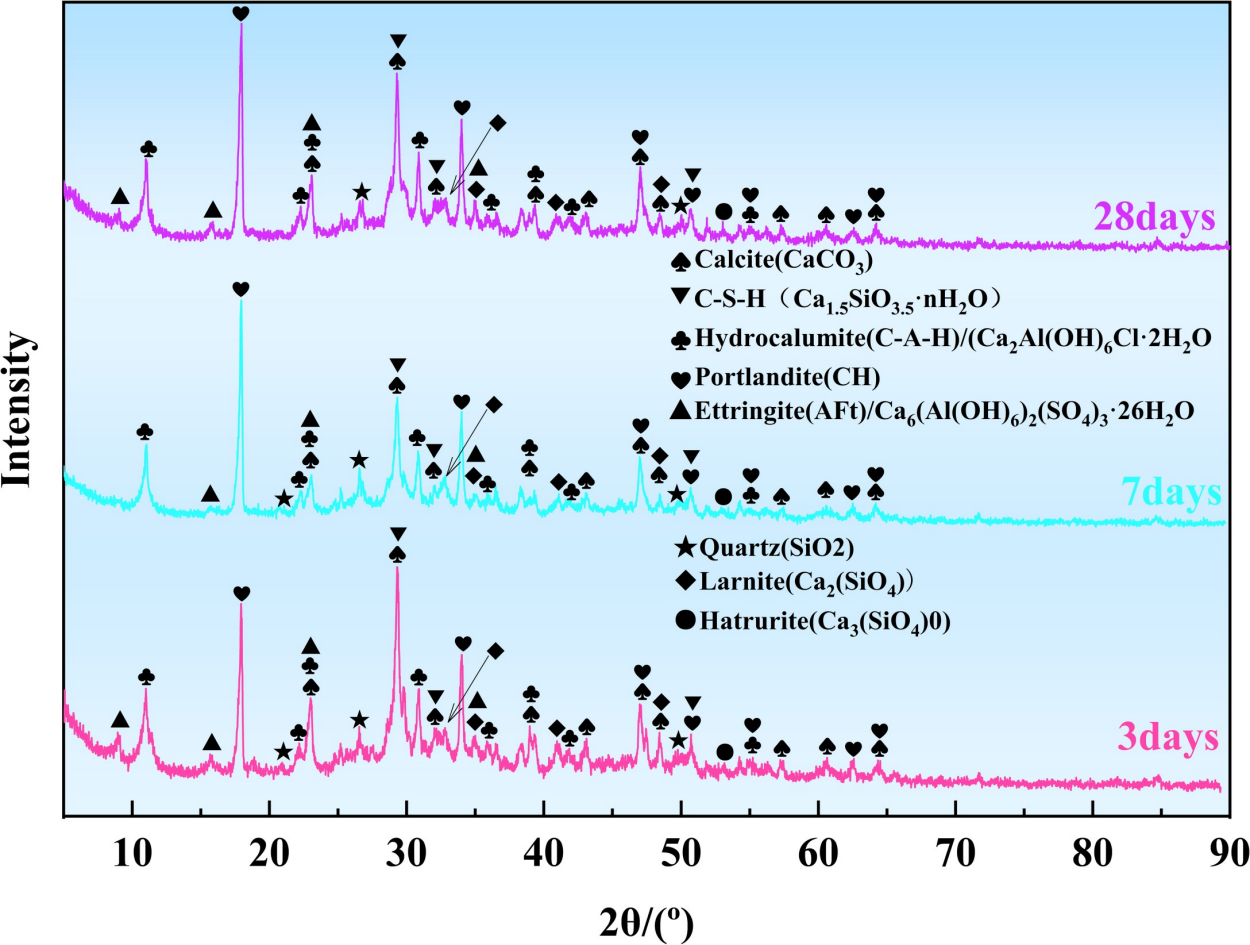
XRD patterns of MUCG at different curing age.

As shown in Fig 7, phases such as CaCO_3_, amorphous C-S-H gel, C-A-H, CH, and AFt were detected in specimens at different ages. Meanwhile, small amounts of hydration by-products Ca_2_(SiO_4_) and Ca_3_(SiO_4_)0 were also detected.

**Fig 8:**
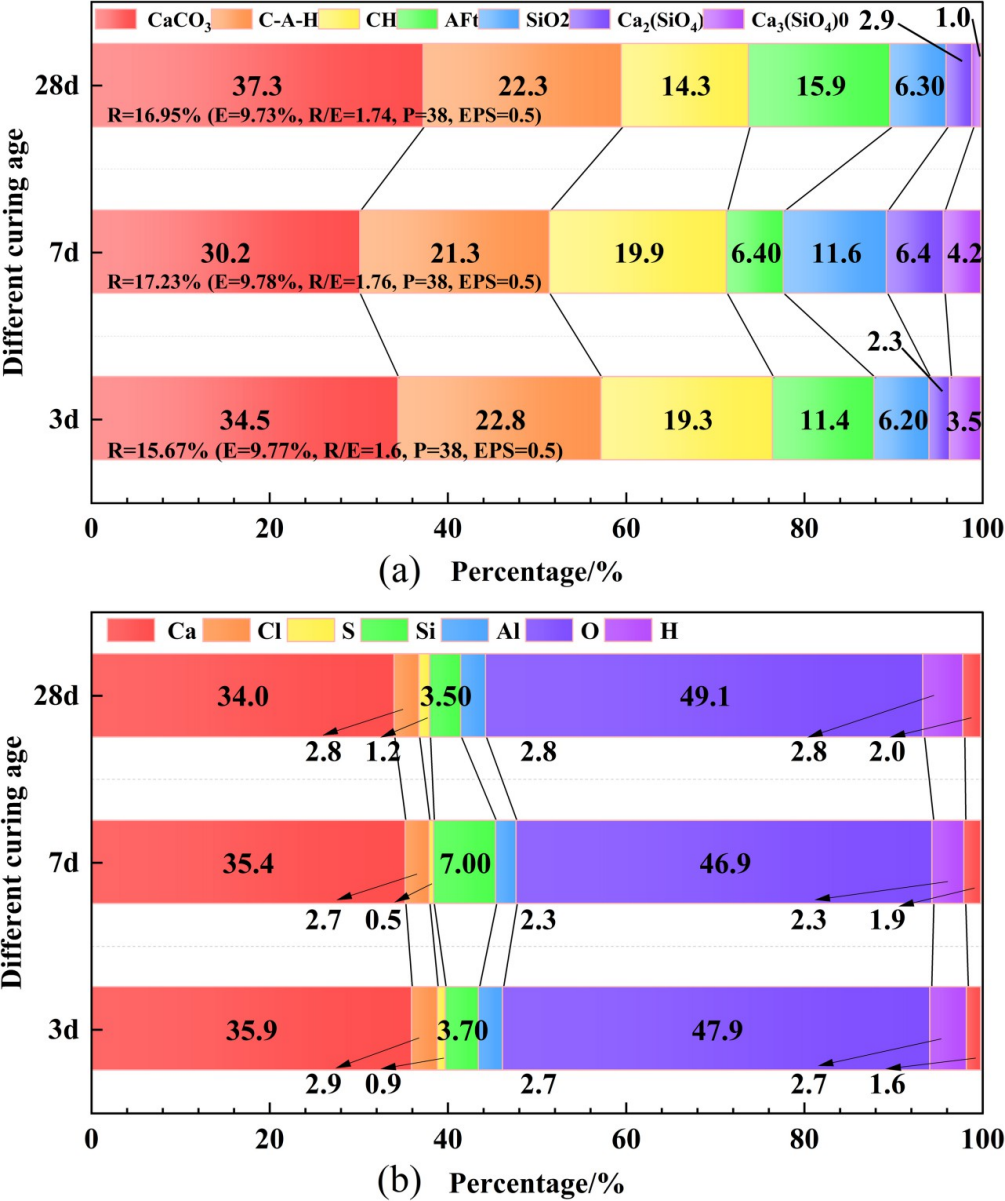
Changes in (a) hydration products and (b) element content patterns of MUCG at different curing ages.

Figs 7 and 8 show that a substantial amount of C-(A)S-H, CH, and AFt was generated between the raw materials in the early stage (up to 3 days). These hydration products filled the pores of the cemented body through chemical and physical bonding, thereby increasing early strength [73], giving rise to the early strength characteristics of MUCGop as described in section 2.4.2.

Fig 8 shows that the changes in hydration product content are not significant across different curing ages. Specifically: (i) The C-A-H content remains stable at around 22%. The CH content is steady at around 19% from Day 3 to Day 7, then decreases to 14.3% from Day 7 to Day 28. The AFt content decreases from 11.4% to 6.4% between Day 3 and Day 7, then increases to 15.9% from Day 7 to Day 28. Amorphous C-S-H was detected, but its content could not be determined through semi-quantitative analysis. Additionally, the CaCO_3_ content decreases from 34.4% to 30.2%, then increases to 37.3% by Day 28.

As seen from Fig 7 (ii) from Day 3 to Day 7, the change in the intensity of CaCO_3_ diffraction peaks (23°, 29°) is not significant, and the calcium content measured by XRF is around 35%, indicating that CaCO_3_ originates from raw materials. The intensity of the CaCO_3_ diffraction peaks increases from Day 7 to 28, attributed to accidental carbonation caused by the reaction of CH with CO_2_ in the atmosphere during microscopic specimen preparation [74] (grinding, screening, storage, and transfer [75, 76]). Additionally, the oxygen content measured by XRF increases from 46.9% to 47.9%, confirming the increase in CaCO_3_ content from Day 7 to 28, as shown in Fig 8. The benefit of cement carbonation lies in its ability to sequester CO_2_ within the cementitious material, thereby reducing carbon emissions [77]. However, it also has a drawback: it reduces the alkalinity of the cement, which can corrode the passivation layer around reinforcing steel and damage the interface between the steel and concrete [78]. It is important to note that a detailed analysis of the advantages and disadvantages of cementitious material carbonation is beyond the scope of this study.(iii) From Day 3 to 7, the change in the intensity of C-S-H diffraction peaks (29°, 32°, 50.6°) is not significant, however, it increases from Day 7 to Day 28. The main peaks of CH diffraction (at 17°, 33°, and 47°) do not change significantly from Day 3 to 7 but weaken on Day 28. Similarly, the diffraction peak of SiO_2_ (at 26.5°) weakens on Day 28. After the initial curing period, a secondary pozzolanic reaction occurs between CH and the SF interface layer, producing a large amount of C-S-H gel. This reaction reduces the number of CH crystal lattices, optimizes the microscopic structure of the cemented body, and enhances its mechanical strength [78]. The intensity of C-A-H diffraction peaks (at 11°, 23°, and 30°) and other weak peaks remains relatively unchanged. In contrast, the intensity of AFt diffraction peaks (at 9°, 15°, 23°, and 34°) increases from Day 7 to Day 28. This is because a large amount of CH is generated in the early stage of cement hydration reaction, a significant part of which dissolves and releases OH⁻, promoting the continuous fracture of Si-O and O-A bonds in the surface glass of raw material (UC, USF, B) particles, generating tetrahedral [SiO_4_]^4-^ and [AlO_4_]^5-^ [79, 80]. At the same time, Ca^2+^, Al^3+^, and SO_4_^2-^ are released and continuously dissolved in the slurry, leading to their increasing concentration in the slurry. Ca^2+^, SO_4_^2-,^ and [AlO_4_]^5-^ were combined to form AFt, while Ca^2+^ and Al^3+^ were combined with [SiO_4_]^4-^ to form C-S-H [81] and C-A-H gel substances. The intensity changes of C-A-H, CH, and AFt diffraction peaks are consistent with the changes in hydration product content (i).

The results of (i) and (iii) demonstrate the strength enhancement from Day 3 to Day 28, and from Day 7 to Day 28 as described in section 2.4.2. The strength experiences a slight decrease from Day 3 to Day 7, and the cause necessitates analysis through changes in porosity. Further detection of the amorphous C-S-H gel is required using FTIR, coupled with observation through SEM. Top of form.

#### 3.5.2 FTIR analysis of hydration products chemical bonds

**Fig 9:**
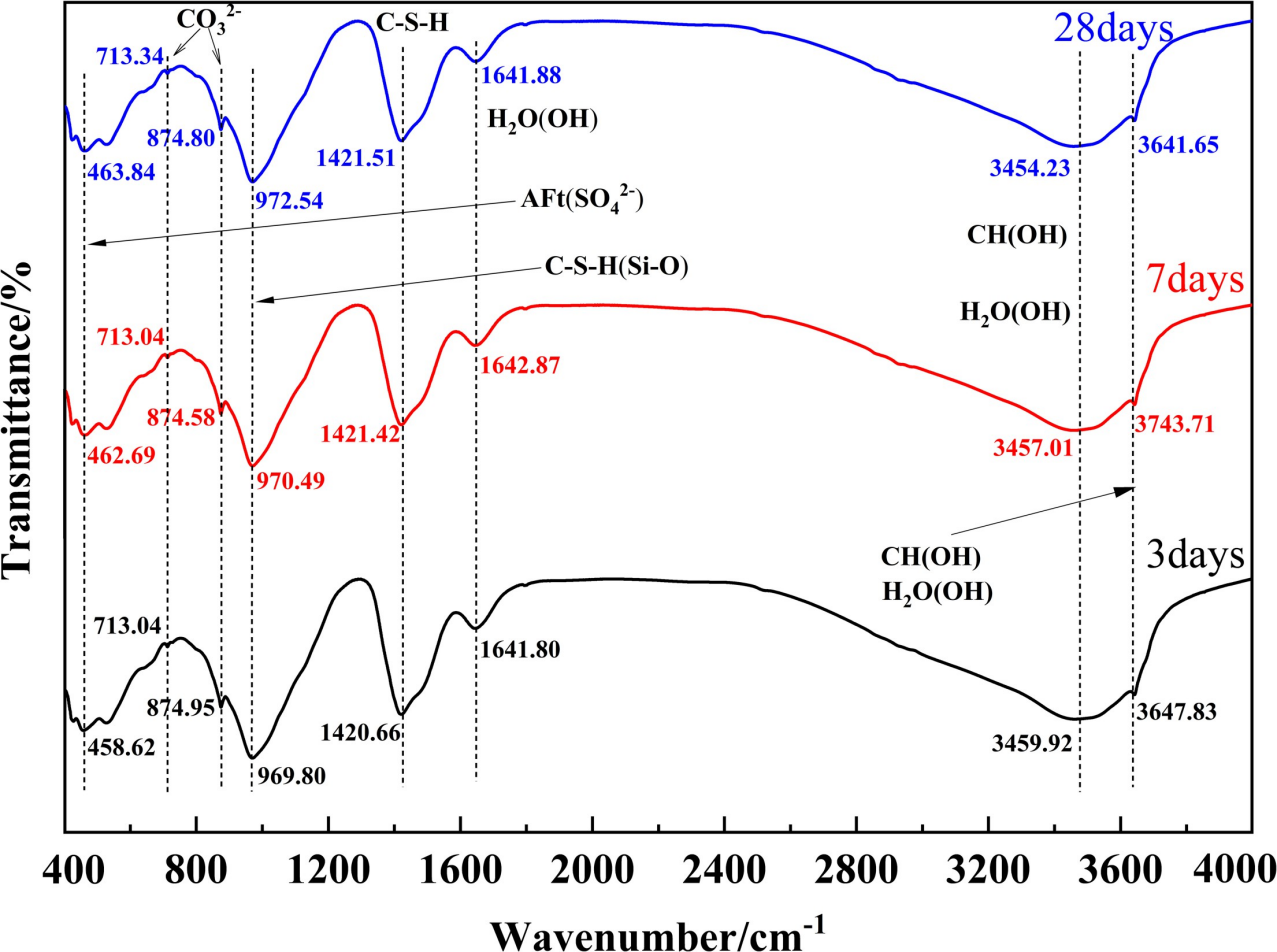
FTIR spectra of MUCG at different curing age.

To further identify the various kinds of hydration products of MUCG, the molecular and bond structural changes of MUCG were analyzed using FTIR. The FTIR spectra profile in Fig 9 can describe the following features:

- Carbonate (CO_3_^-2^) absorption peaks near 713 cm^-1^ (in-plane vibration) and 874 cm^-1^ (out-of-plane vibration) correspond to calcite (polycrystalline CaCO₃). The carbonate vibration peak increases continuously till Day 28, further confirming its association with CH carbonization [74].
- Absorption peaks in the range of 3640–3750 cm^-1^ are characteristic of -OH absorption peaks of Ca(OH)_2_ [82].
- The bands observed in the range of 900–1100 cm^-1^ are related to the in-plane bending vibration of Si-O bonds in C-S-H [83, 84], with significant C-S-H gel characteristic absorption peaks appearing near 1421 cm^-1^ [85]. The broad bands in the range of 3100–3600 cm^-1^ and around 3741 cm^-1^ can be attributed to the OH stretching of bound water in C-S-H and C-S-H [84].
- Absorption peaks in the range of 443–464 cm^-1^ correspond to the absorption bands of SO_4_^2-^ in AFt [85, 86], whereas the absorption peaks near 1641 cm^-1^ and 3457 cm^-1^ respectively correspond to the stretching and bending absorption of crystalline water in ettringite, further confirming the presence of AFt [85]. The results further indicate that the primary hydration products of MUCG are C-(A) S-H gel, CH, and AFt.

#### 3.5.3 BET porosity analysis

**Fig 10:**
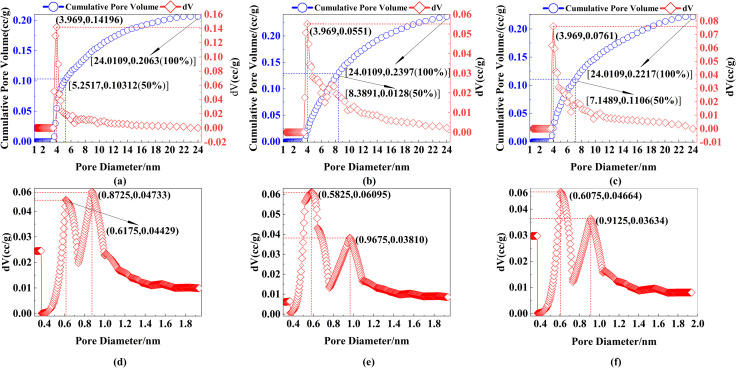
Pore size distribution of MUCG under different CA: mesopores on (a) Day 3, (b) Day 7, and (c) Day 28; micropores on (d) Day 3, (e) Day 7 and (f) on Day 28.

This study adhered to the pore size classification standard established by J. Rouquerol et al. [87], which categorizes pores with diameters less than 2 nm as micropores, those between 2 and 50 nm as mesopores, and those exceeding 50 nm as macropores. Employing the DFT method and the HK method, the pore distributions of mesopores and micropores within the cemented body were determined, as depicted in Fig 10 Across the three different curing ages, mesopores (ranging from 2 to 24 nm) constituted over 90% of the pore space, followed by micropores at nearly 10%, while macropores were nearly absent. These findings suggest that MUCGop showcases a relatively compact structure and enhanced strength.

The specific surface area of the cemented body (mesopores + micropores) was measured by multi-point BET. The surface areas were estimated to be 80.618 m^2^/g, 78.680 m^2^/g, and 68.701 m^2^/g for Days 3, 7, and 28 respectively. Compared to Day 3, the specific surface areas of Day 7 and Day 28 decreased by 2.4% and 14.8%, respectively. From Day 7 to Day 28, the specific surface area decreased by 12.7%.

(1) Mesopores

Examining from the perspective of 100% cumulative pore volume, median pore size, and most probable pore size:

- The 100% cumulative pore volumes for the three curing ages are 0.2063 cc/g, 0.2397 cc/g, and 0.2217 cc/g, respectively. This indicates a 16.2% increase from Day 3 to Day 7, followed by a 7.5% decrease from Day 7 to Day 28.
- The median pore volumes for the three curing ages are 5.2517 cc/g, 8.3891 cc/g, and 7.1489 cc/g, respectively. These findings suggest an overall increasing trend in median pore size with curing time, with a growth of 60% from Day 3 to Day 7 and a subsequent decrease of 14.7% from Day 7 to Day 28.
- As seen from Fig 10(A)–10(C), the most probable pore size for the three curing ages is 3.969 nm, corresponding to pore volumes of 0.14196 cc/g, 0.0551 cc/g, and 0.0761 cc/g. Compared to Day 3, the pore volume on Day 7 and Day 28 decreased by 61.2% and 46.4%, respectively, while from Day 7 to Day 28, the pore volumes increased by 38.1%.
  (2) Micropores

Analyzing from the perspective of most probable pore size and mode pore size:

- As depicted in Fig 10(D)–10(F), two prominent peaks are evident for the micropore volumes across three different curing ages. While the left peak remains relatively stable, the right peak displays a noticeable decline in micropore volume as curing time increases, specifically decreasing by 13.4% from Day 3 to Day 7, 17.9% from Day 3 days to Day 28, and 4.6% from Day 7 to Day 28. These findings suggest that the curing process effectively restrains the development of micropores, resulting in a reduction in their number and the densification of the micropore structure [88], thereby continuously enhancing the compressive strength of the cemented body.
- The mode pore sizes measured by the HK method show mode pore sizes of 0.872 nm, 0.583 nm, and 0.608 nm for Day 3, 7, and 28, respectively. Compared to Day 3, the mode pore sizes decreased by 33.1% and 30.7% for Day 7 and Day 28, respectively, while from Day 7 to Day 28, there was a 4.3% increase.

In summary, the results for specific surface area and micropores (most probable pore size) indicate a gradual decrease in the overall pore volume of both mesopores and micropores. At different curing ages, the pore volume of mesopores (100% cumulative pore volume, median pore size) initially increases and then decreases, which aligns with the observed slight decrease in strength from Day 3 to Day 7 and the slight increase in strength from Day 7 to Day 28 described in section 2.4.2. On the other hand, the pore volume of mesopores (most probable pore size) and micropores (mode pore size) first decreases and then increases, which contradicts the same trend in strength. The specific reasons for these changes in porosity and strength require further investigation and discussion regarding the surface morphology of MUCGop.

#### 3.5.4 Micro-morphological analysis of hydration products by SEM

Figures depicts the SEM micrographs of MUCG samples at different curing ages. No obvious cracks are observed, and crystal material growth is evident, indicating high mechanical strength.

**Fig 11:**
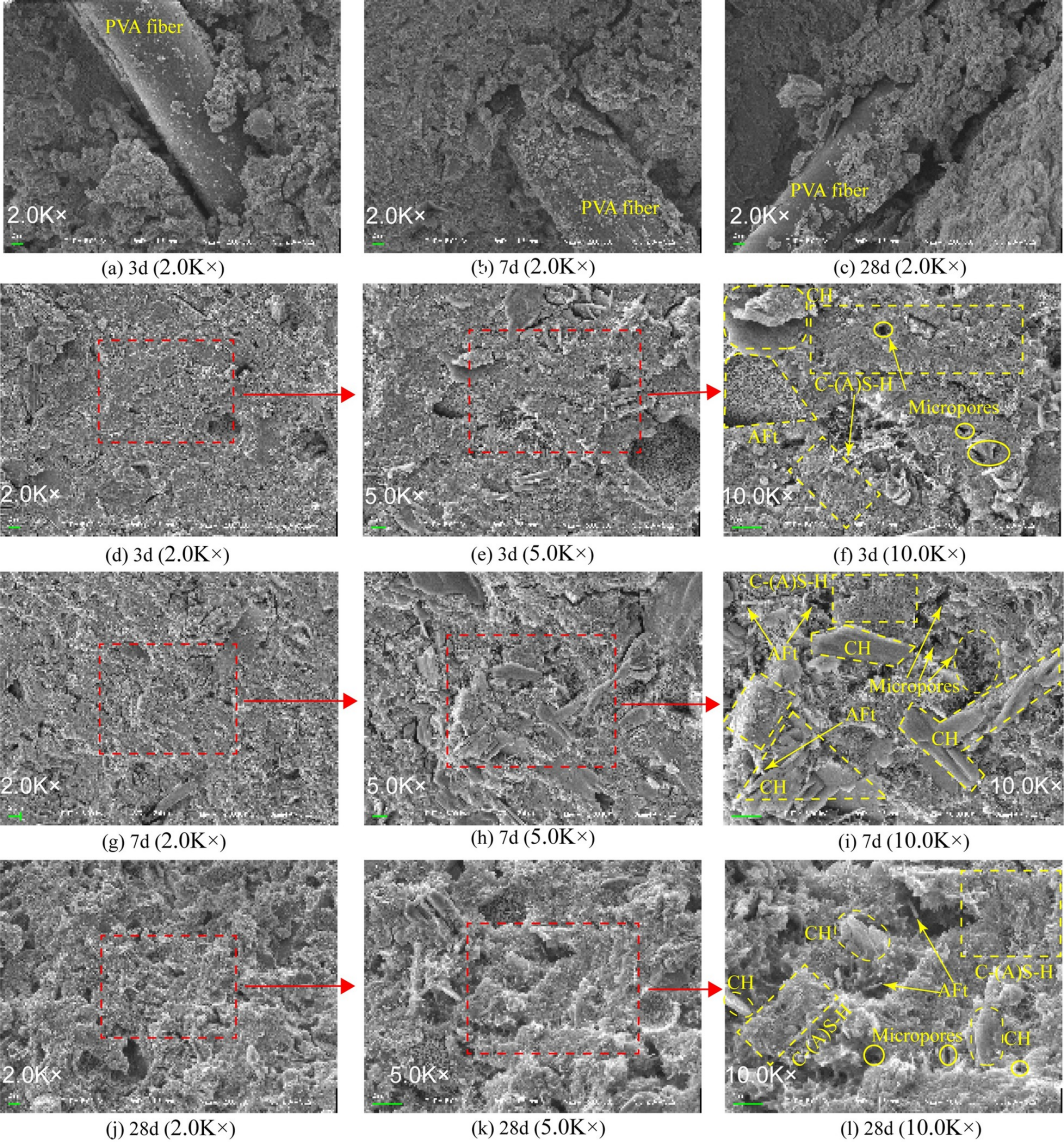
Micro-morphology of MUCG samples under different CA (2.0K×∼10.0K×): (a) Day 3, (b) Day 7, (c) Day 28, (d∼f) Day 3, (g∼i) Day 7 and (j∼l) Day 28.

Fig 11(A)–11(C) reveals that with prolonged curing time, aggregate structures are formed between PVA fibers (columnar) and hydration products of the raw materials, continuously filling the interface gaps, and thus enhancing the mechanical strength of the cemented body.

(i) Fig 11 shows that under 2.0K× magnification, the substrate surfaces of (d) Day 3 and (g) Day 7 are smooth, whereas that of (j) Day 28 exhibits a rough surface. (ii) Under 5.0K× magnification, the crystal development and substrate density of (j) Day 28 are more pronounced compared to (e) Day 3 and (h) Day 7. No cracks are observed in (e) Day 3 and (k) Day 28 specimens, while slight cracks appear in (h) Day 7, corresponding to a slight decrease in strength from Day 3 to Day 7. This decrease in strength may be due to the flow of unutilized silica fume, which has a relatively high content of 8% in this study, within the tortuous capillary network. This flow creates a significant driving force and increases mesopore volume, leading to shrinkage of the cemented body. [89, 90]. When micro-cracks develop in the material, high stresses concentrate at their tips, while relatively lower stress levels are present around them. This stress concentration induces deformation and local swelling at the crack tips, ultimately compromising the material’s structural integrity [91]. By further adjusting the content of silica fume, bentonite, and PVA fibers, as well as the diameter and length of PVA fibers [4], the micro-crack formation can be suppressed [92], thus enhancing the overall mechanical strength of the material. It is important to note that this study focuses on the overall performance of the slurry, and as such, achieving optimal strength alone is not feasible. By 28d, the consumption of silica fume decreases, and the shrinkage induced by silica fume disappears, with more C-S-H gel filling the microcracks. (ii) demonstrates the intrinsic reasons for the slight decrease in strength from Day 3 to Day 7 as discussed in section 2.4.2.

(iii)Under 10.0K× magnification, Fig 11(F), 11(i), and (l) reveal that the main interaction between AFt, CH, and C-(A) S-H gel involves small fibrous and flocculent crystals, forming a relatively complete substrate network structure [93]. In Fig 11(F), AFt is aggregated in a lamellar shape and surrounded by CH and C-(A) S-H gel. The needle-like AFt in Fig 11(I) corresponding to Day 7 and 11 (l) corresponding to Day 28 is more developed. CH in Fig 11(F) (Day 3) and 11(i) (Day 7) exhibits a voluminous layered morphology, while in Fig 11 (I) (Day 28), it appears as small voluminous flakes. The C-(A) S-H gel in Fig 11 (I) (Day 28) is more developed compared to Fig 11(H) (Day 3) and 11(i) (Day 7). The bonding between CH, AFt, and C-(A) S-H gel is not strong at Days 3 and 7, indicating their relative independence. By Day 28, the more developed CH, AFt, and C-(A) S-H gel are uniformly and strongly bonded through chemical or mechanical interlocking, resulting in a more compact and dense structure. This strong bonding is more conducive to strength development than the hydrogen bonds between voluminous layered blocks [94]. This in turn explains the observed small increase in strength from Day 7 to Day 28.

(i), (ii), and (iii) are in good agreement with the XRD results for the change in content of hydration products as discussed in section 2.5.1.

## 4 Conclusions and limitations

In this study, additives such as silica fume, bentonite, PVA fibers, superplasticizers, and accelerators were used to enhance ultrafine cement. Through orthogonal experiments, a modified ultrafine cement-based grouting material (MUCG) was developed. The optimal formulation was determined by analyzing the significant influencing factors using range analysis and multivariate linear regression theory. XRD, FTIR, BET, and SEM techniques were employed for microscopic testing and analysis of MUCGop to investigate its hydration mechanism. The main conclusions of this study are as follows:

(1) The results demonstrate a high consistency in the significance of factors identified in MLRA and range analysis: The fluidity of the slurry is most significantly influenced by the superplasticizer, followed by silica fume and bentonite. The superplasticizer also has the greatest impact on viscosity, with other factors having less noticeable effects. For the water bleeding rate, the superplasticizer remains the most significant factor, followed by bentonite. The setting time is primarily affected by the setting accelerator, with the superplasticizer also playing a significant role. Finally, compressive strength is most significantly influenced by silica fume, followed by PVA fibers.
(2) The optimal composition of MUCG comprises 9% silica fume, 0.2% bentonite, 0.3% PVA fibers, 0.15% superplasticizer, and 2% accelerator. This formulation exhibits excellent overall performance, including flowability (262 mm), low viscosity (24.53 s), a water bleeding rate of 1% (significantly below the 5% threshold), moderate setting time, and high compressive strength (Day 3, 17.58 MPa), making it ideal for grouting projects that demand high injectability and strength.
(3) MUCG generates a large amount of C-(A)S-H gel, CH, and AFt in the early hydration period (Day 3), revealing the early strength characteristics of MUCGop. At different ages, the content of C-A-H stabilizes at 22%; CH stabilizes at 19% on Day 7 and remains stable at 14% by Day 28. AFt decreases from 11.4% to 6.4%, then rises to 15.9% by Day 28. The diffraction peak of the amorphous C-S-H gel shows a minimal change from Day 3 to Day 7, then increases by Day 28, indicating an improvement in strength from Day 3 to Day 28, and from Day 7 to Day 28. XRD and FTIR analyses together confirm unexpected carbonation on Day 28, with the CaCO_3_ content rising to 37.3%.
(4) The results for specific surface area and micropores (most probable pore size) indicate a gradual decrease in total porosity. Over different curing periods, mesopores (100% cumulative pore volume, median pore size) initially increase and then decrease. SEM observations reveal the appearance of microcracks in the cemented body on Day 7, correlating with a slight decrease in strength from Day 3 to Day 7. Additionally, SEM analysis shows more developed needle-shaped AFt, C-(A)S-H gel, and small-volume plate-like CH at later stages, with uniformly high degrees of cementation and denser structures. This development explains the slight strength increase from Day 7 to Day 28. The changes in the matrix structure of the cemented body and morphology of hydration products observed in SEM at different ages align with XRD findings on the variations in hydration product content.

## 5 Limitations

This study determines the composition of MUCG through laboratory experiments; however, actual engineering applications may require adjustments to the MUCG composition and other grouting parameters based on varying geological conditions and grouting equipment factors. The models y6 and y7 in this study may exhibit nonlinearity. Future research could benefit from employing regression fitting with more accurate response surface methods or gene expression programming models, combined with machine learning techniques. Such an approach could enable real-time optimization of MUCG composition tailored to specific engineering conditions. This would involve collecting rock samples on-site, employing CT scanning to obtain parameters such as fracture length, width, and roughness as accurately as possible, and evaluating MUCG performance during actual grouting operations.

## Supporting information

S1 Dataset (XLSX)
